# Non-targeted Colonization by the Endomycorrhizal Fungus, *Serendipita vermifera*, in Three Weeds Typically Co-occurring with Switchgrass

**DOI:** 10.3389/fpls.2017.02236

**Published:** 2018-01-09

**Authors:** Prasun Ray, Yingqing Guo, Jaydeep Kolape, Kelly D. Craven

**Affiliations:** Noble Research Institute, LLC, Ardmore, OK, United States

**Keywords:** grass endophyte, microcosm, mycorrhiza, *Panicum virgatum*, Rhizobox, *Sebacina*

## Abstract

*Serendipita vermifera* (=*Sebacina vermifera*; isolate MAFF305830) is a mycorrhizal fungus originally isolated from the roots of an Australian orchid that we have previously shown to be beneficial in enhancing biomass yield and drought tolerance in switchgrass, an important bioenergy crop for cellulosic ethanol production in the United States. However, almost nothing is known about how this root-associated fungus proliferates and grows through the soil matrix. Such information is critical to evaluate the possibility of non-target effects, such as unintended spread to weedy plants growing near a colonized switchgrass plant in a field environment. A microcosm experiment was conducted to study movement of vegetative mycelia of *S. vermifera* between intentionally inoculated switchgrass (*Panicum virgatum* L.) and nearby weeds. We constructed size-exclusion microcosms to test three different common weeds, large crabgrass (*Digitaria sanguinalis* L.), Texas panicum (*Panicum texanum* L.), and Broadleaf signalgrass (*Brachiaria platyphylla* L.), all species that typically co-occur in Southern Oklahoma and potentially compete with switchgrass. We report that such colonization of non-target plants by *S. vermifera* can indeed occur, seemingly via co-mingled root systems. As a consequence of colonization, significant enhancement of growth was noted in signalgrass, while a mild increase (albeit not significant) was evident in crabgrass. Migration of the fungus seems unlikely in root-free bulk soil, as we failed to see transmission when the roots were kept separate. This research is the first documentation of non-targeted colonization of this unique root symbiotic fungus and highlights the need for such assessments prior to deployment of biological organisms in the field.

## Introduction

The *Serendipitaceae* (formerly Sebacinales Group B) belong to a taxonomically, ecologically and physiologically diverse group of fungi in the Basidiomycota (kingdom Fungi). While historically recognized as orchid mycorrhizae, recent ITS based phylogenetic studies have demonstrated both their pandemic distribution and the broad spectrum of mycorrhizal types they form (Warcup, 1988; Suarez et al., 2008; Oberwinkler et al., 2013). Indeed, ecological studies using PCR-based detection methods have found *Serendipitaceae* in field specimens of bryophytes (mosses), pteridophytes (ferns) and all families of herbaceous angiosperms (flowering plants) from temperate, subtropical and tropical regions. These natural host plants include, among others, liverworts, wheat, maize and *Arabidopsis thaliana*, a genetic model plant traditionally viewed as non-mycorrhizal (Warcup, 1988; DeMars and Boerner, 1996; Selosse et al., 2002). *Serendipita vermifera* exemplifies this phenotype, and though it was originally isolated from an Australian orchid (Warcup, 1988), we have successfully established beneficial interactions between this fungal species and many different experimental hosts, including the model plant *Arabidopsis* (Ray and Craven, 2016). Considering their proven beneficial effects on switchgrass growth (Ghimire and Craven, 2011; Ray et al., 2015) and their apparent ubiquity (Weiss et al., 2011), *Serendipitaceae* fungi should be considered as a previously hidden, but amenable and effective microbial tool for enhancing plant productivity and stress tolerance.

Switchgrass (*Panicum virgatum* L.) has been identified by the United States Department of Energy as an important bio-feedstock for cellulosic ethanol production (McLaughlin and Kszos, 2005). A large part of the rationale behind its selection relates to an astonishingly deep root system that enhances nutrient and water uptake, as well as stabilizes the soil matrix and fixes carbon, both ecological services that promote its utility on marginal landscapes (Ma et al., 2000). Although productive and competitive with minimal inputs once established, weed competition can impede establishment success and resulting productivity (Schmer et al., 2006; Mitchell et al., 2008) of switchgrass. Unfortunately, this extensive root system is also an Achilles heel in the first year of cultivation (establishment), as most productivity goes to root production and little is allocated to the shoots. Co-occurring annual broadleaf weeds are common in the establishment of switchgrass, and if populations are dense, establishment can be reduced (Renz et al., 2009).

As weed competition is indeed the most common cause of establishment failure in switchgrass, and *Serendipitaceae* fungi appear to have an extraordinarily broad host range, the possibility for un-intended colonization of weedy species in the vicinity of inoculated switchgrass plants exists. Therefore, we sought to document the incidence of non-targeted colonization of commonly co-occurring weeds when growing alongside a switchgrass colonized with *S. vermifera.* Toward this end, we conducted two unique greenhouse based microcosm studies to investigate the ability of *S. vermifera* to colonize non-targeted crops via movement through a root-free bulk soil or via an interconnected network of roots likely present in a majority of natural and agricultural plant communities. We used switchgrass as our intended target of inoculation and three different competing weeds, large crabgrass (*Digitaria sanguinalis*), Texas panicum (*Panicum texanum*) and broadleaf signalgrass (*Brachiaria platyphylla*), as “bait” for testing non-target effects. Each of these weeds was chosen as they are known to be typically present in the Southern Oklahoma region (Miller et al., 1975; Blount et al., 2003) and potentially compete with switchgrass (Kering et al., 2013). This research would not only inform efficient weed management strategies when *Serendipitaceae* fungi are utilized, but also weighs in on the decision to deploy non-native microbes in a broader sense.

## Materials and Methods

### Plant Material

Seeds of large crabgrass (*Digitaria sanguinalis*), Texas panicum (*Panicum texanum*), broadleaf signalgrass (*Brachiaria platyphylla*) and switchgrass (*Panicum virgatum* L.), cultivar Alamo were procured from The Noble Research Institute, LLC. All the seeds were briefly surface sterilized following the protocol modified after Xi et al. (2009). Briefly, seeds were washed with 50% Clorox^®^ (8.25% sodium hypochlorite, Clorox, Oakland, CA, United States) containing 0.1% TWEEN^®^20 (Amresco, Solon, OH, United States) for 30 min. After five washes with sterile water, seeds were soaked in sterile water and kept in 4°C overnight. Subsequently, seeds were treated one additional time with 50% Clorox^®^ for 30 min, washed with sterile water for five times and air dried on sterile filter paper. Surface sterilized seeds were sown in sterile Metro-Mix^®^ 360 (Sungro Horticulture, Agawam, MA, United States) and maintained in the green house for germination under standard conditions (temperature range, 26–29°C) with 50% relative humidity under a 16:8 h photoperiod. Four week-old uniform size seedlings were used for all subsequent experiments.

### Preparation of Inoculum Using Bentonite Clay as a Carrier

The *S. vermifera* strain MAFF305830 used in this study was obtained from the National Institute of Agro-biological Sciences, Tsukuba, Japan. Clay particle based inoculum of *S. vermifera* was prepared as described in Ray et al. (2015). The clay particles were thoroughly sieved using mesh size 10 (2 mm) to get a uniform particle size. One-liter media bottles were filled with 400 ml of clay particles by volume and 150 ml of Modified Melin Norkan’s (MMN) (Marx, 1969) media pH 7. The clay particles and MMN media were mixed homogeneously by shaking, and then sterilized by autoclaving. Each bottle was inoculated with 50 ml of a 4-week-old *S. vermifera* liquid culture prepared in 250-ml Erlenmeyer flasks. An equivalent amount of MMN broth was added to a control set of bottles used as mock inoculum in this study. Both the mock and the inoculated bottles were incubated in a slanted, stationary position at 24°C for 8 weeks. The bottles were agitated once per week for uniform distribution of the inoculum.

### Preparation of the Microcosm Assemblies

**FIGURE 1:**
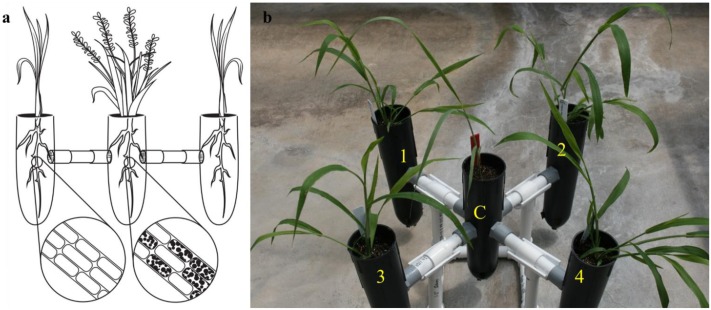
The exocosm assembly to investigate the movement of *S. vermifera* between switchgrass and competing weeds mediated by vegetative mycelia. **(a)** Illustration of an exocosm assembly. The colonization of individual root cells at the end of the study by *S. vermifera* are represented by black dots (enlarged) in the illustration. **(b)** Five single-cell root trainers were connected by 16 cm long PVC pipes. The openings of the four connector pipes were covered with fine nylon mesh from either side to exclude roots but allow the movement of the finer fungal mycelia. Switchgrass in the center (C) was colonized with *S. vermifera* (MAFF305830). Weeds in the four corners (1∼4) were not colonized.

**FIGURE 2:**
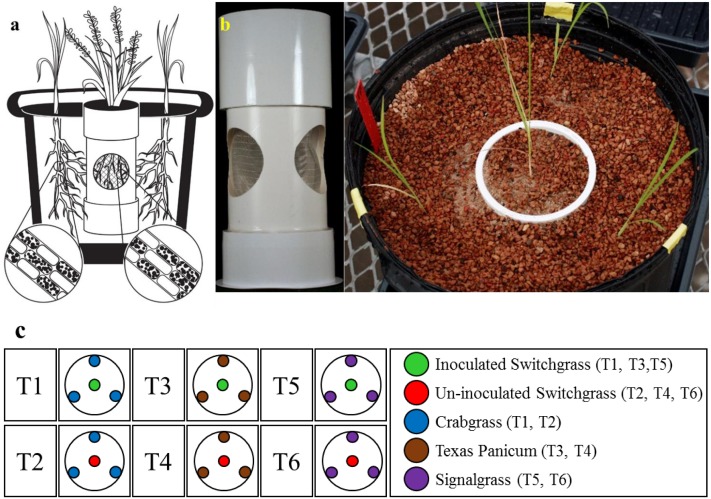
The endocosm assembly to investigate the movement of *S. vermifera* between switchgrass and competing weeds mediated by vegetative mycelia. **(a)** An illustration of the endocosm assembly. The colonization of individual root cells at the end of the study by *S. vermifera* is represented by black dots (enlarged). **(b)** An actual endocosm experimental unit. Three circular openings were cut in a pipe. The openings were covered with fine nylon mesh to exclude the roots but allow the movement of fungal mycelia. The bottom of the pipe was sealed with a perforated base to allow water movement. This was placed in the center of 5 gallon pot. Switchgrass in the center was inoculated with *S. vermifera*. Three weeds planted in the periphery were un-inoculated. **(c)** The experimental design for the endocosm study. T1, T3, T5: Switchgrass in the center was inoculated with *S. vermifera*. T2, T4, T6: Switchgrass in the center was un-inoculated. T1, T2: Crabgrass in the periphery, T3, T4: Texas Panicum in the periphery, T5, T6: Signalgrass in the periphery.

To investigate controlled, non-targeted colonization, we created two unique microcosm assemblies developed at Noble Research Institute, LLC as illustrated in **Figures 1, 2**. As the plant compartments are externally connected, we designated the first microcosm (**Figure 1**) assembly as an “exocosm.” Conversely, as all plants in the second microcosm assembly (**Figure 2**) were placed inside a single pot, around a central compartment, we named this assembly an “endocosm.” The exocosm was designed to evaluate the ability of *S. vermifera* to colonize co-occurring weeds in a *root free* environment, and the endocosm assembly was designed to evaluate the ability of *S. vermifera* to colonize co-occurring weeds via co-mingled switchgrass and weed root systems.

For construction of the exocosm, five D25L single-cell root trainers (7 cm × 25 cm | diameter × height, Stuewe & Sons, Inc., Tangent, OR United States) were used and interconnected by short PVC pipes (2 cm × 16 cm | diameter × length) (**Figure 1b**). The openings of the four connector pipes were covered with fine nylon mesh from either side to exclude roots but allow the movement of the finer fungal mycelia (**Figure 1a**). Both cones and the connectors were filled with Metro-Mix^®^ 360 potting mixture.

For the endocosm assembly, three circular openings (6 cm diameter) were created in a pipe (10 cm × 23 cm | diameter × height) as shown in **Figure 2b**. The openings were covered with fine nylon mesh (**Figure 2a**) to exclude the roots but allow the movement of fungal mycelia. The bottom of the pipe was sealed with a perforated base for water passage. This assembly was placed in the center of a 5 gallon pot and filled with Turface^®^ MVP^®^ (Buffalo Grove, IL, United States).

### Experimental Set Up

In the exocosm assembly (**Figure 1b**), a switchgrass seedling was transplanted in the central root trainer cell (C) and inoculated with *S. vermifera* using bentonite clay following the protocol developed in our laboratory (Ray et al., 2015). The four peripheral compartments (1, 2, 3, and 4) of each exocosm were transplanted with un-inoculated weed seedlings of a single type. Separate exocosms were maintained for each of the three weed species. The experiment was conducted in triplicate and repeated twice in The Noble Research Institute’s greenhouse facility.

The endocosm study was conducted using the assembly as described in **Figure 2**. A switchgrass seedling was transplanted in the central compartment and colonized with *S. vermifera* using bentonite clay. Three un-colonized weed seedlings were planted in the periphery, outside of the central compartment (**Figures 2b,c**). A similar setup with an un-inoculated switchgrass seedling in the center was also maintained as a control for each weed species. In all, the experimental design consisted of six treatments (**Figure 2c**), with four replicates per treatment. The experimental units were arranged in a randomized complete block design.

For both studies, the plants were maintained in the greenhouse for 8 weeks post transplanting, under standard conditions (temperature range, 26–29°C) with 50% relative humidity under a 16:8 h photoperiod. Plants were watered 2∼3 times per week.

### Verification of the Colonization of Non-targeted Weeds by *S. vermifera*

**FIGURE 3:**
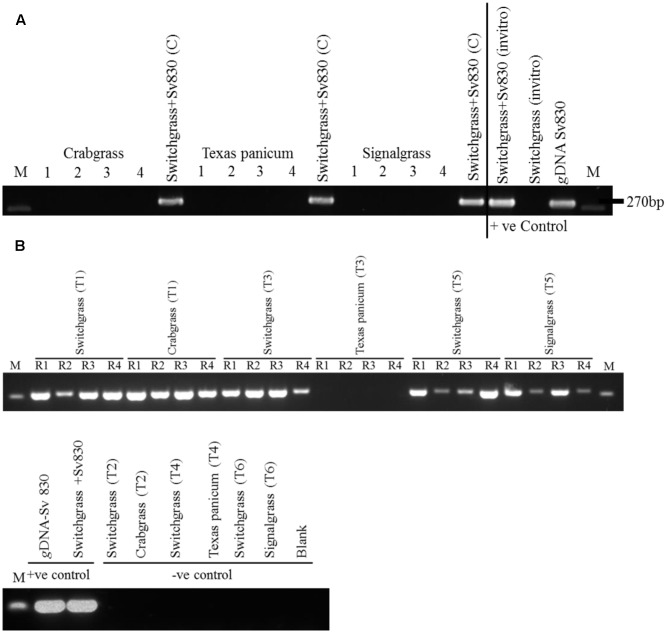
**(A)** Verification of root colonization by *S. vermifera* in switchgrass and in the four peripheral weeds in the exocosm assembly. Sv830: *S. vermifera* (MAFF305830), M: Marker; 1∼4: four peripheral un-inoculated weeds; Switchgrass+Sv830 (C): Inoculated switchgrass in the center. **(B)** Verification of root colonization by *S. vermifera* in switchgrass and in the peripheral weeds in the endocosm assembly. Sv830: *S. vermifera* (MAFF305830), M: Marker; R1∼R4: Four replicate seedlings. T1, T3, T5: Switchgrass in the center was inoculated with *S. vermifera*. T2, T4, T6: Switchgrass in the center was un-inoculated. T1, T2: Crabgrass in the periphery, T3, T4: Texas Panicum in the periphery, T5, T6: Signalgrass in the periphery. Colonization was confirmed by nested PCR of switchgrass and weed root DNA by *Serendipita* specific primers. *g*DNA from *S. vermifera* pure culture, *in vitro* germinated switchgrass seedling colonized with or without *S. vermifera* respectively were used as controls for PCR assay.

After 8 weeks, the colonization by *S. vermifera* was confirmed by nested PCR using *S. vermifera* (MAFF305830) specific primers (Ray et al., 2015). *g*DNA was isolated from inoculated and mock inoculated switchgrass and weed roots. The 3′ region of the 18S (SSU), ITS1 and ITS2, the 5.8S ribosomal subunit and the 25-28S (LSU) were amplified by direct PCR using NSSeb1 and NLSeb1.5R. Direct PCR amplified a ∼2.2 kb fragment. Subsequently, the primary PCR product was diluted to 1:200 and used as a template for a nested PCR using ITS3Seb and ITS3Seb-R primers covering the 5.8S-coding sequence and highly variable ITS2 region of *S. vermifera* ribosomal DNA (rDNA) (**Figure 3**).

Further, in the endocosm study where the two different rooting systems (switchgrass and weeds) were separate, but still in very close proximity (**Figure 2a**), the possibility of mixing between weed roots and switchgrass roots during harvesting was eliminated by running PCR on weed root DNA using switchgrass-specific primer *PvCon1* (Supplementary Figure S1 and Supplementary Table S1).

### Effect of Non-targeted Colonization on the Biomass of Co-occurring Weeds

In instances where the PCR screen revealed a co-occurring weed that was apparently infected by *S. vermifera*, the aboveground material was harvested for estimation of biomass to evaluate the effect of non-targeted colonization of *S. vermifera* on the biomass of co-occurring weeds.

### Data Analysis

**FIGURE 4:**
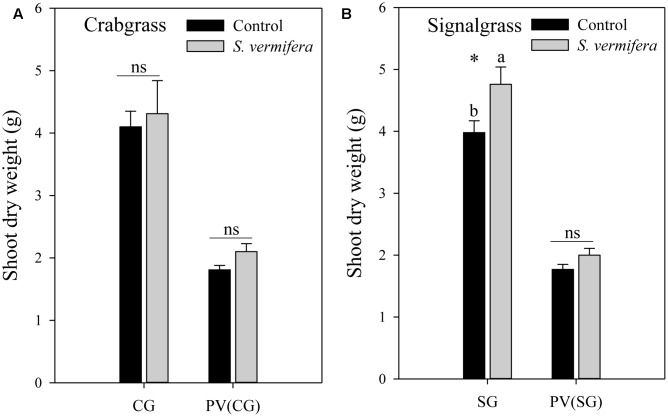
Effect of non-targeted colonization of *S. vermifera* on biomass of **(A)** Crabgrass and **(B)** Signalgrass planted around central colonized or un-colonized control switchgrass in the endocosm assembly (**Figure 2c**) 8 weeks after colonization. Error bars denote standard error of mean. ns: *p* > 0.05. CG: Crabgrass; SG: Signalgrass; PV(CG) and PV(SG): Switchgrass (*Panicum virgatum*) in the center surrounded by Crabgrass (CG) and Signalgrass (SG) respectively.

The data were analyzed using the Analysis of Variance (ANOVA) method. When a significant *F*-test was observed, treatment means were compared using least significant difference (LSD) at *p* < 0.05 using CoStat statistical software 6.4 (Cohort Berkeley, CA, United States). The results were plotted graphically (**Figure 4**) using SigmaPlot 12.5 (Systat Software, San Jose, CA, United States).

### *In Vitro* Colonization of Host Plants by *S. vermifera*

Colonization of switchgrass, signalgrass, and crabgrass roots by *S. vermifera* was initially confirmed by a PCR based assay (**Figure 3B**). Additionally, to obtain more direct evidence and to demonstrate that fungus does indeed penetrate the host root, we colonized switchgrass, signalgrass and crabgrass *in vitro* with *S. vermifera*.

Seeds of all three host plants were surface sterilized following the protocol modified after Xi et al. (2009) as described earlier. Sterilized seeds were placed onto large square petri dishes (120 mm, Greiner Bio-One North America Inc., Monroe, NC, United States) containing M media (Bécard and Fortin, 1988; Schultze, 2013), pH 5.5, and amended with 0.3% Phytagel^TM^ (MilliporeSigma, St. Louis, MO, United States). Petri dishes were incubated in a Conviron^®^ (Winnipeg, MB, Canada) plant growth chamber for 7 days at 24°C with 60% relative humidity under 16:8 h photoperiod for seed germination.

For *in vitro* colonization, inoculum of *S. vermifera* was prepared by grinding 50 mg (fresh wt. basis) of 2 weeks old vegetative mycelia in 500 μl of M media. After seed germination, individual seedlings were inoculated by putting a drop (10 μl) of inoculum with a sterile pipette on the media in close proximity (∼3 cm) to the roots. Inoculated seedlings were then incubated in the Conviron^®^ plant growth chamber for two additional weeks. After 2 weeks in the Conviron^®^, plants were harvested and the roots were subsequently processed for staining and microscopy.

### Fluorescence Staining and Confocal Microscopy

*S. vermifera*-colonized root samples from switchgrass, signalgrass, and crabgrass were fixed in 50% (v/v) ethanol for 1 h at room temperature immediately after harvesting (Bravo et al., 2016). Fixed root samples were then cleared with 20% (v/v) KOH for 30 min at 50°C. To visualize fungal vegetative mycelia, fixed and cleared roots were stained with 10 μg/ml aqueous solution of WGA-AF 488^®^ (Life Technologies, Carlsbad, CA, United States) (Javot et al., 2007) overnight at room temperature. To visualize the plant cell wall, roots were then counter stained with 10 μg/ml aqueous solution of propidium iodide (PI: Biotium, Hayward, CA, United States) (De Smet et al., 2008; Pumplin et al., 2010) for 30 min at room temperature. Root samples were subsequently washed three times with distilled water in-between each chemical treatment. Root samples were finally cut into 1 cm long pieces and mounted onto glass slides using distilled water as the mounting media. Slides were transferred onto the stage of an inverted (inverted objective lenses) Leica TCS-SP8 point scanning confocal microscope (Leica Microsystems, Wetzlar, Germany) equipped with a 40x water-immersion objective lens. Fungal hyphae stained with WGA-AF 488^®^ were excited using 488-nm of the White Light Laser (WLL), and emission was detected at 493–535 nm. Plant cell walls stained with PI were visualized by exciting at 538 nm by WLL, and emission was detected at 592-650 nm. Sequential scanning between lines was used to detect WGA-AF 488 emission from fungal hyphae and PI from plant cell walls. Images were captured using the Leica TCS-SP8 running LAS X software (Leica Microsystems, Wetzlar, Germany), and images from these channels were overlaid to show fungal colonization in plant cells.

## Results

### Colonization of Switchgrass and Co-occurring Weeds by *S. vermifera*

After 8 weeks, *S. vermifera* colonization in switchgrass and in the peripheral weeds were checked by nested PCR using *S. vermifera* specific primers (Supplementary Table S1). In the exocosm study, none of the three different types of weeds in the four peripheral cones were colonized by *S. vermifera* (**Figure 3A**). Further, we were unable to successfully amplify the *S. vermifera*-specific band from potting mix collected from the pipes connecting the cones, suggesting that either mycelia do not move freely through the inter-connected pipes or their relative abundance was beyond detectable limits by PCR. Conversely, in the endocosm study we found that crabgrass and signalgrass plants in the periphery were indeed colonized by *S. vermifera* when planted around a central, colonized switchgrass plant, while Texas Panicum remained un-infected (**Figure 3B**). Further, this “weed root DNA” was not contaminated by switchgrass root DNA, suggesting that very close proximity between the roots of the intended switchgrass host and those of nearby Crabgrass or Signalgrass weeds can result in unintended colonization of the latter.

### Non-targeted Colonization by *S. vermifera* Can Influence the Biomass of Weeds

Having established that non-targeted colonization is possible, we evaluated whether it lead to biomass gains. Indeed in the endocosm, a mild increase in biomass of both crabgrass (ns) and signalgrass (^∗^*p* < 0.05) was observed when planted around a central colonized switchgrass plant, in comparison to those planted around the un-inoculated control (**Figure 4**). Although crabgrass and signalgrass were colonized in a non-targeted manner through seemingly co-mingled root systems when planted around colonized switchgrass, (**Figure 3B**), such non-targeted colonization did not contribute to a significant increase in biomass of crabgrass (**Figure 4A**) with respect to un-inoculated cohorts (*p* > 0.05). However, increase in biomass was significant in the case of signalgrass (**Figure 4B**) (^∗^*p* < 0.05). No significant increase in biomass was observed in switchgrass planted in the center, surrounded either by crabgrass or by signalgrass respectively. To summarize, although there was a trend of enhanced biomass when *S. vermifera* was present, both for the switchgrass and co-occurring crabgrass and signalgrass weeds, the increase in biomass was significant in case of signalgrass only. Besides this, it was also evident that, the biomass of competing weeds for the same period were almost twice as much as switchgrass (**Figures 4A,B**).

### Visualization of *S. vermifera* Colonization by Confocal Microscopy

**FIGURE 5:**
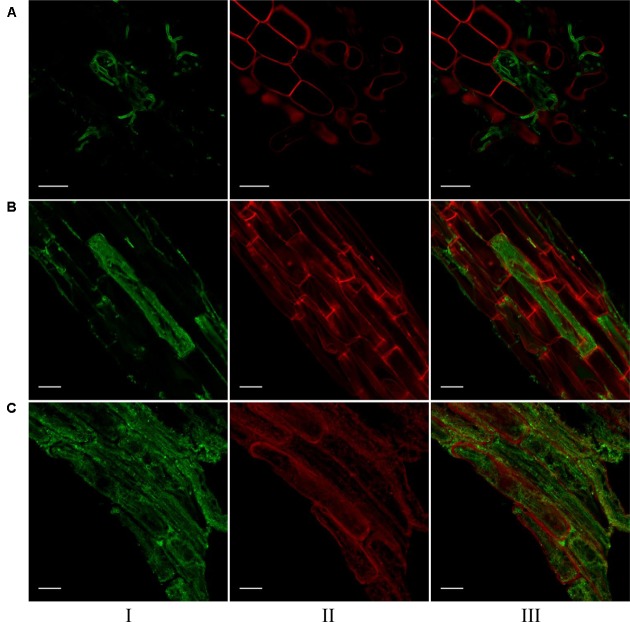
Visualization of *S. vermifera* colonized root cells of **(A)** switchgrass, **(B)** signalgrass, and **(C)** crabgrass by confocal microscopy. Fungal hyphae stained with WGA-AF 488^®^ were visualized in green channel (I). Plant cell wall stained with PI were visualized in red channel (II). Images from these channels were overlaid (III) to show fungal colonization in plant cells. Bar = 20 μm.

Confocal microscopic images of root cells of switchgrass, signalgrass and crabgrass colonized by *S. vermifera* are presented in **Figure 5**. *S. vermifera* root colonization is typically marked by sparse growth between cells and occasional cells containing dense intracellular packing of fungal hyphae. Interestingly, the extent of intracellular colonization in the three different grass species was also visually distinct, suggesting that the degree of colonization by *S. vermifera* may vary with plant host species.

## Discussion

There is currently no evidence that fungi in the *Serendipitaceae* can traverse any significant distance in the soil matrix and colonize nearby root systems. However, strains of this fungus (including the one tested herein) do form associations with hosts that initiates with plant recruitment of the fungus from the surrounding soil, suggesting that such movement is possible. Further, the movement of fungal mycelia through the soil matrix does occur naturally for other fungi such as *Phialocephala* spp. (Sieber and Grünig, 2006). Garnica et al. (2013) have even suggested the possibility of co-localized host plants being linked in a common mycelial network. A similar hypothesis has been suggested by Ryberg et al. (2009), who determined that dominating ectomycorrhizal fungi, including members of the *Serendipitaceae*, were shared by *Dryas octopetala* and *Salix reticulata* in an alpine cliff ecosystem in Northern Sweden. As our ultimate objective is to deploy Serendipitoid fungi as plant growth-promoting agents in the field, we performed this study to clarify the ability of one such strain (*S. vermifera* MAFF305830) to move within the soil matrix, such as occurs in other fungal groups (Taylor and Bruns, 1997; Vincenot et al., 2008).

In the exocosm study, we report that none of the three different type of weeds planted in the peripheral compartments were colonized by *S. vermifera* (**Figure 3A**). As the design of the exocosm restricts root growth to the central compartment, these findings suggest that mycelium of the Serendipitoid symbiont cannot traverse roughly 16cm of soil in 8 weeks. Conversely, in the endocosm study, we found that crabgrass and signalgrass in the periphery were colonized by *S. vermifera* when planted surrounding the central pre-colonized switchgrass (**Figure 3B**). In contrast to the exocosm, the endocosm allows both switchgrass and the weed root systems to share the same space, separated only by a fine nylon membrane (**Figure 2a**). Thus, it would seem that one reason for the inability of *S. vermifera* to colonize peripheral weeds in the exocosm could be the rate of mycelial extension over time. However, since PCR with soil DNA in the connector region was also negative, we cannot substantiate this hypothesis. In a separate field study where wheat plants inoculated with this fungus are grown in a plot alongside un-inoculated plants at one foot (30.48 cm) intervals, we also fail to find “new colonization events” where an initially fungus-free plant becomes colonized over the growing season (∼8 months) (data not presented). From these data, it would seem that migration of this strain of *S. vermifera* is fairly limited, even in an initially sterile growth medium where, presumably, competition is not a factor.

Incidence of non-targeted colonization by *S. vermifera* significantly improve biomass of signalgrass but not crabgrass in our study. However, we failed to observe the typical significant (*p* < 0.05) biomass gains we would expect in switchgrass (Ghimire and Craven, 2011; Ray et al., 2015), likely due to the low number of replicates (three) included in this study. Besides this, the biomass of competing weeds for the same period were almost twice as much as switchgrass (**Figure 4**). This could be another reason behind non-significant biomass increase in switchgrass when *Serendipita* was present. Our objective essentially focused on migration of the symbiont, but to address the question of whether this fungus can significantly improve biomass of these weedy species would require additional study.

As those of us in the scientific community working with these *Serendipitaceae* fungi have thus far been unable to identify a non-host, assessing the impact of introducing this symbiont to any native rhizosphere is highly relevant. Herein we provide evidence that *S. vermifera* can indeed migrate through the interconnected network of roots of co-occurring plant species resulting in un-intended colonization of non-targeted plants. However, such migration is very limited, and we hypothesize that new infections would likely be rare events.

Further, unlike crabgrass and signalgrass, Texas panicum was not colonized by *S. vermifera*. This suggests the possibility that this plant is not an effective host for this strain of *S. vermifera*, a hypothesis we are currently following up on.

The undesired colonization of non-target crops by any plant growth promoting microbe is likely a real phenomenon that merits serious cause for caution and study. It is our opinion that the utilization of native strain(s) of such microbes, including *S. vermifera*, is the approach with perhaps the fewest negative consequences. Further, native strains are presumable adapted to the particular agro-climatic region they are intended for, thus enhancing their potential for persistence and effectiveness, while ameliorating issues related to weed management and regulation. Our laboratory has recently isolated the first North American strain of *Serendipita*, named *Serendipita vermifera* ssp. *bescii*, from the roots of a switchgrass plant (Ray and Craven, US patent pending). Using native microorganisms should diminish the potential for invasiveness or other adverse non-target effects on the native soil microbiota. Hence, *S. bescii* could be of special interest for application to the fields of the Southern Great Plains of United States in the future.

## Author Contributions

PR and YG carried out all the experiments. JK conducted the confocal microscopy. PR and KC planned the experiments, analyzed the data and wrote the article.

## Conflict of Interest Statement

The authors declare that the research was conducted in the absence of any commercial or financial relationships that could be construed as a potential conflict of interest.
