# Supplementary material for: Non-targeted Colonization by the Endomycorrhizal Fungus, Serendipita vermifera, in Three Weeds Typically Co-occurring with Switchgrass
Source: Front Plant Sci. 2018 Jan 9;8:2236. doi: 10.3389/fpls.2017.02236 (PMC5767251; doi:10.3389/fpls.2017.02236)
Supplement: Supplementary file 1 [file Data_Sheet_1.docx]

**Supplementary Table**

**Table S1** List of primers used for the present study

| **Marker name** | **Primer sequence** 5'🡪3' | **Reference** |
| --- | --- | --- |
| *NSSeb1* | cttcttagagggactgtcagga | Weiß *et al*. (2011) |
| *NLSeb1.5R* | attcgctttaccgcacaaggc | Garnica et al. (2013) |
| *ITS3Seb* | gcatcgatgaagaacgcagc | Mary Berbee group |
| *ITS3Seb-R* | gagaccaaactccggtgaaa | Ray et al. (2015) |
| *NL4* | ggtccgtgtttcaagacgg | O’Donnell (1993) |
| *PvAct68-F* | ctgcttcaaccctagaagagagcgtgcaagtagcagaa | This study |
| *PvAct68-R* | tgtttccattcttgcagattgcagatcattcaacataa | This study |
| *PvAct36-F* | gtaaatgcaataattggtacaataatatatgtgatgac | This study |
| *PvAct36-R* | taactgatacccaaccttttgcagagtacctgtaggaa | This study |
| *PvCon1-F1* | tagcagtacctagcaaaggt | This study |
| *PvCon1-R1* | atgtggagtaccgctgctt | This study |
| *PvCon2-F1* | gcttccaaaccctagcgcag | This study |
| *PvCon2-R1* | aatatgacaatcaggagtat | This study |

**Supplementary Figure**

**Figure S1.** Assessment of switchgrass specific primers used for this study. (a) PvCon1; (b) PvCon2; (c) PvAct68; (d) PvAct36. R1~R2: Replicate assays**
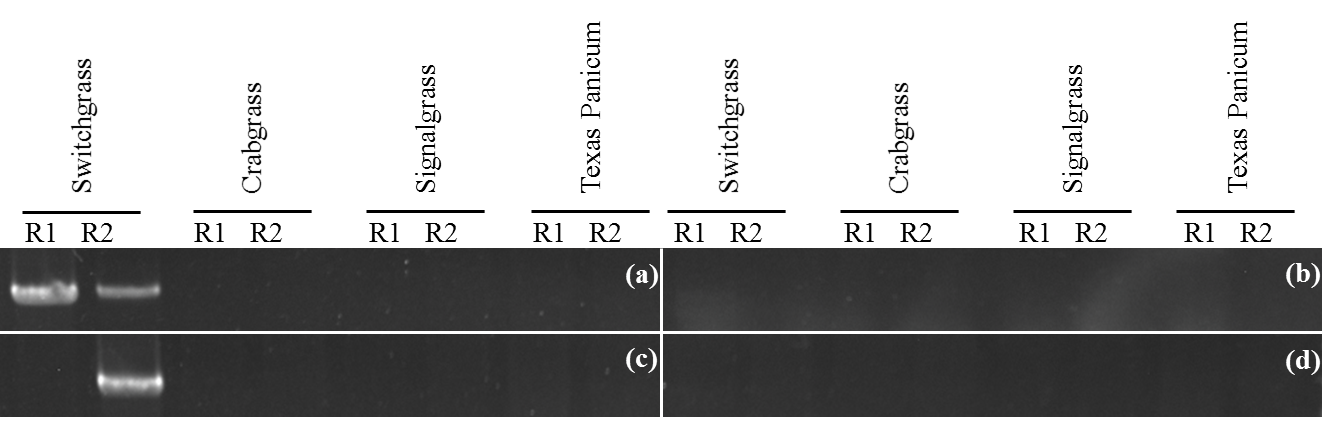
**
